# MLML: consistent simultaneous estimates of DNA methylation and hydroxymethylation

**DOI:** 10.1093/bioinformatics/btt459

**Published:** 2013-08-21

**Authors:** Jianghan Qu, Meng Zhou, Qiang Song, Elizabeth E. Hong, Andrew D. Smith

**Affiliations:** Molecular and Computational Biology, University of Southern California, Los Angeles, CA 90089, USA

## Abstract

**Motivation:** The two major epigenetic modifications of cytosines, 5-methylcytosine (5-mC) and 5-hydroxymethylcytosine (5-hmC), coexist with each other in a range of mammalian cell populations. Increasing evidence points to important roles of 5-hmC in demethylation of 5-mC and epigenomic regulation in development. Recently developed experimental methods allow direct single-base profiling of either 5-hmC or 5-mC. Meaningful analyses seem to require combining these experiments with bisulfite sequencing, but doing so naively produces inconsistent estimates of 5-mC or 5-hmC levels.

**Results:** We present a method to jointly model read counts from bisulfite sequencing, oxidative bisulfite sequencing and Tet-Assisted Bisulfite sequencing, providing simultaneous estimates of 5-hmC and 5-mC levels that are consistent across experiment types.

**Availability:** http://smithlab.usc.edu/software/mlml

**Contact:** andrewds@usc.edu

**Supplementary information:** Supplementary material is available at *Bioinformatics* online.

## 1 INTRODUCTION

DNA methylation is an important epigenetic mark in mammals. In addition to the extensively studied 5-methylcytosine (5-mC) modification, its oxidation product, 5-hydroxymethylcytosine (5-hmC), has been observed at substantial levels in both somatic and embryonic stem cells (Kriaucionis and Heintz, 2009; Tahiliani *et al.*, 2009). Recent studies of 5-hmC in mouse TET knock-out models (Ito *et al.*, 2010), mouse zygotic development (Iqbal *et al.*, 2011) and multiple cell types (Globisch *et al.*, 2010; Ito *et al.*, 2011; Kinney *et al.*, 2011; Sun *et al.*, 2013) suggest that 5-hmC is involved in epigenetic regulation.

The current most comprehensive and accurate method for profiling cytosine methylation is bisulfite sequencing (BS-seq). Treatment with sodium bisulfite converts unmethylated cytosines to uracils, but does not distinguish between 5-mC and 5-hmC (Huang *et al.*, 2010), and consequently the yield of methylation from BS-seq is the sum of 5-mC and 5-hmC levels. Two recently developed techniques, oxidative bisulfite sequencing (oxBS-seq) (Booth *et al.*, 2012) and Tet-Assisted Bisulfite sequencing (TAB-seq) (Yu *et al.*, 2012), provide high-throughput single-base resolution measurements of 5-mC and 5-hmC, respectively. Any two of BS-seq, TAB-seq or oxBS-seq can be combined to profile both the 5-mC and 5-hmC methylomes of a cell population, and especially when studying 5-hmC, proper interpretation of results depends on having some estimate of the 5-mC level. However, naive manipulation of read count frequencies from independent sequencing experiments often produces two kinds of ‘overshoot’ problems in estimating 5-mC and 5-hmC levels. When combining BS-seq with TAB-seq, the 5-mC level at a given CpG site can be estimated by subtracting the 5-hmC level (TAB-seq) from the combined 5-mC + 5-hmC level (BS-seq). The result can be negative, because of random sampling (or systematic error) in each experiment. Similarly, combining TAB-seq and oxBS-seq could lead to estimates of 5-mC and 5-hmC levels exceeding 100%. These overshoot sites may constitute a substantial proportion. In one dataset based on oxBS-seq technology, 17% of CpG sites captured by reduced representation bisulfite sequencing (RRBS) and oxRRBS experiments exhibited overshoot (Booth *et al.*, 2012). To fully leverage the information in these data requires some method for making consistent estimates of 5-mC and 5-hmC levels.

We present maximum likelihood methylation levels (MLML) for simultaneous estimation of 5-mC and 5-hmC, combining data from any two of BS-seq, TAB-seq or oxBS-seq, or all three when available. Our estimates are consistent in that 5-mC and 5-hmC levels are non-negative, and never sum over 1. In an important subset of cases, our estimates are not only consistent but also show significantly greater accuracy at sites with lower coverage.

## 2 METHODS

Each of BS-seq, TAB-seq and oxBS-seq provides some amount of information about both the 5-mC and 5-hmC levels. Our approach is to combine information from any pair or all three of these experiments, and arrive at maximum likelihood estimates (MLEs) for the 5-mC and 5-hmC levels. A similar method has been developed in the context of haplotype frequency estimation from pooled sequencing (Kessner *et al.*, 2013). To explain our method, we assume the data are from TAB-seq and BS-seq experiments for the same biological sample. The more general formulation is provided in Supplementary Information.

Focusing on an individual CpG site, let *p_m_* denote the methylation level (a probability), *p_h_* the hydroxymethylation and 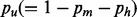 the level of unmethylated C. In the TAB-seq experiment, let *h* denote the number of C reads mapping over the CpG site, and let *g* denote the T reads mapping over the same CpG. The total reads covering the CpG site in the TAB-seq experiment is then *h* + *g*. Similarly, let *t* denote the number of C reads mapping over the site in the BS-seq experiment, whereas *u* denotes the number of T reads, and the total reads covering the CpG in the BS-seq experiment is 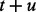. If values for *p_m_* and *p_h_* are known, *h* and *u* are binomial random variables, i.e. 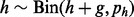, and 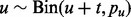:

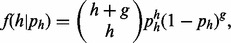


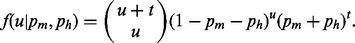

Given observations of 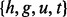, when no overshoot would result, we use the frequencies to estimate 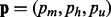. In this case, the frequencies directly give MLEs. At overshoot sites, we introduce latent variables and use expectation maximization to approximate the MLE for **p**. Let 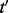 (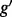) be the number of C (T) reads in BS-seq (TAB-seq) that correspond to 5-mCs. Then 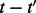 (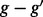) is the number of C (T) reads corresponding to 5-hmC (unmethylated C). The complete data likelihood is then

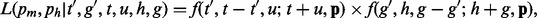

where 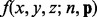 is a multinomial p.m.f. Estimates for *p_h_* and *p_m_* are then computed by expectation maximization algorithm to account for the latent 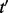 and 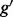 (Supplementary Information). The MLEs can be compared with binomial confidence intervals around corresponding frequency estimates if direct readouts (e.g. for 5-hmC in the case of TAB-seq) are available. When estimates fall outside the specified confidence interval, sites are flagged as being ‘strongly’ inconsistent. An overabundance of such sites might suggest systematic error.

## 3 RESULTS

**Fig. 1.**
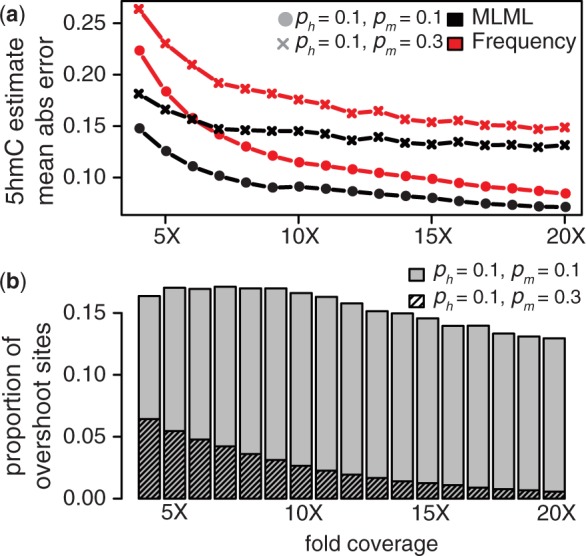
Accuracy is improved at lower coverage using MLML (BS-seq + TAB-seq). (**a**) Average absolute errors of 5-hmC level estimates at overshoot sites. (**b**) Proportion of overshoot sites in simulated data

To understand the properties of our estimators and the frequency method, we used simulations with fixed coverage and precisely set levels for 5-mC and 5-hmC, assuming the experiments were BS-seq and TAB-seq. The case of BS-seq and oxBS-seq is symmetric with the estimates for *p_h_* and *p_m_* exchanged. For each valid combination of 5-mC and 5-hmC levels from 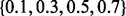, we simulated from binomial distributions for both BS-seq and TAB-seq. Estimates for *p_h_* and *p_m_* were made using the maximum likelihood method and the frequency method, which estimate *p_h_* using 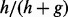 and *p_m_* using 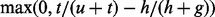. The relative error (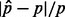) for both estimation methods was computed and then averaged over 100 000 simulations for each parameter combination. The average estimation errors are presented in Supplementary Table S1. Estimates of *p_h_* are more accurate using MLML, especially at lower values of *p_h_* and low coverage. For example, when the true values are 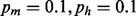, the MLML reduces the average relative error by >23% at overshoot sites compared with frequency estimates when the coverage is 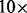, and this reduction in error increases to 57% for such sites covered only 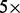. The trend for errors of *p_h_* estimates is shown in Figure 1a, indicating the accuracy advantage for MLML as a function of coverage. The simulation also revealed substantial amounts of overshoot sites under different 5-mC and 5-hmC level combinations (Fig. 1b, Supplementary Tables).

## Supplementary Material

Supplementary Data
