# Supplementary material for: MLML: consistent simultaneous estimates of DNA methylation and hydroxymethylation
Source: Bioinformatics. 2013 Aug 21;29(20):2645–6. doi: 10.1093/bioinformatics/btt459 (PMC3789553; doi:10.1093/bioinformatics/btt459)
Supplement: Supplementary Data [file supp_29_20_2645__index.html]

MLML: Consistent simultaneous estimates of DNA methylation and hydroxymethylation — MLML: consistent simultaneous estimates of DNA methylation and hydroxymethylation — MLML: consistent simultaneous estimates of DNA methylation and hydroxymethylation — Supplementary Data 

# MLML: consistent simultaneous estimates of DNA methylation and hydroxymethylation

## Supplementary Data

files

**Files in this Data Supplement:**

- Supplementary Data - pdf file
- Supplementary Data - tex file
