# Supplementary material for: MLML: consistent simultaneous estimates of DNA methylation and hydroxymethylation
Source: Bioinformatics. 2013 Aug 21;29(20):2645–6. doi: 10.1093/bioinformatics/btt459 (PMC3789553; doi:10.1093/bioinformatics/btt459)
Supplement: Supplementary Data [file supp_btt459_document_supp.pdf]

1 DERIVATIONS FOR EM ALGORITHM FOR MLE

We present here a full model that combines read counts from oxBS-seq, TAB-seq and BS-seq experiments to estimate 5mC and 5hmC levelst at single CpG resolution. Estimation using only two out of the three experiments is a special case of the full model, and can be achieved by setting corresponding read counts to 0 for the unavailable experiment. Let p_m be the probability/level of methylation, and p_h that of hydroxymethylation, at a particular CpG site. Let the total number of reads (C+T) covering the CpG site in TAB-seq experiment be $h+g$, $t+u$ in BS-seq, and $m+l$ in oxBS-seq, where h, t, m are the number of C reads, and g, u, l are the number of T reads in corresponding experiments. Conditional on p_m and p_h, m, h and u are binomial random variables, $m \sim \text{Binomial}(m+l, p_m)$, $h \sim \text{Binomial}(h+g, p_h)$, and $u \sim \text{Binomial}(t+u, 1-p_m-p_h)$. Hence,

$$f(m|p_m) = \binom{m+l}{m} p_m^m (1-p_m)^l, \tag{1}$$

$$f(h|p_h) = \binom{h+g}{h} p_h^h (1-p_h)^g, \tag{2}$$

$$f(u|p_m, p_h) = \binom{u+t}{u} (1-p_m-p_h)^u (p_m+p_h)^t. \tag{3}$$

1.1 Complete data likelihood

To infer the MLE of p_m and p_h from observations of $\{h, g, u, t, m, l\}$, two latent variables are introduced. Let t' be the number of C reads from 5mCs in BS-seq, and g' be the number T reads from 5mCs in TAB-seq. Thereby, $t' \in \{0, 1, \ldots, t\}$, and $t - t'$ would be the number of C reads from hmCs. Similarly, $g' \in \{0, 1, \ldots, g\}$, and $g - g'$ would be the number of T reads from unmethylated Cs in TAB-seq. The complete likelihood function is thus

$$\begin{aligned} L(p_m, p_h|t'=k, g'=j, m, l, t, u, h, g) = &\binom{m+l}{m} p_m^m (1-p_m)^l \times \binom{t+u}{k, t-k, u} p_m^k p_h^{t-k} (1-p_m-p_h)^u \\ &\times \binom{h+g}{h, j, g-j} p_m^j p_h^h (1-p_m-p_h)^{g-j}. \end{aligned}$$

1.2 Conditional distribution of latent variables

The conditional distribution of t' and g' given the parameter (methylation levels) and observations (read counts) is a binomial distribution with p.m.f.

$$\Pr(t'=k|t, p_m, p_h) = \binom{t}{k} p_m^k p_h^{t-k} (p_m+p_h)^{-t},$$

and

$$\Pr(g'=j|g, p_m, p_h) = \binom{g}{j} p_m^j (1-p_m-p_h)^{g-j} (1-p_h)^{-g}.$$

1.3 E-step

Let $p_m^{(i)}, p_h^{(i)}$ be the estimates of methylation and hydroxymethylation levels at the ith iteration. The auxiliary function is thus

$$\begin{aligned} &Q(p_m, p_h; p_m^{(i)}, p_h^{(i)}) \\ &= \sum_{k=0}^{t} \sum_{j=0}^{g} \log L(p_m, p_h|t'=k, g'=j, m, l, t, u, h, g) \cdot p(t'=k|t, p_m^{(i)}, p_h^{(i)}) p(g'=j|g, p_m^{(i)}, p_h^{(i)}) \\ &= \sum_{k=0}^{t} \sum_{j=0}^{g} \frac{\binom{t}{k}\binom{g}{j} p_m^{(i)k} p_h^{(i)t-k} (1-p_m^{(i)}-p_h^{(i)})^{g-j} p_m^{(i)j}}{(p_m^{(i)}+p_h^{(i)})^t (1-p_h^{(i)})^g} \times [\log(\binom{m+l}{m}\binom{t+u}{k, t-k, u}\binom{h+g}{h, g-j, j}) \\ &\quad + (m+k+j)\log(p_m) + l\log(1-p_m) + (t-k+h)\log(p_h) + (u+g-j)\log(1-p_m-p_h)] \\ &= \sum_{k=0}^{t} \sum_{j=0}^{g} \beta_{ijk} [(m+k+j)\log(p_m) + l\log(1-p_m) + (t-k+h)\log(p_h) + (u+g-j)\log(1-p_m-p_h)] + C \end{aligned} \tag{4}$$

where

$$\beta_{ijk} = \frac{\binom{t}{k}\binom{g}{j} p_m^{(i)j+k} p_h^{(i)t-k} (1 - p_m^{(i)} - p_h^{(i)})^{g-j}}{(p_m^{(i)} + p_h^{(i)})^t (1 - p_h^{(i)})^g},$$

and

$$C = \sum_{k=0}^{t} \sum_{j=0}^{g} \log\left(\binom{m+l}{m}\binom{t+u}{k,t-k,u}\binom{h+g}{h,g-j,j}\right)$$

is a constant.

1.4 M-step

$$\frac{\partial Q}{\partial p_m} = \sum_{k=0}^{t} \sum_{j=0}^{g} \beta_{ijk}[\frac{m+k+j}{p_m} - \frac{l}{1-p_m} - \frac{u+g-j}{1-p_m-p_h}] = 0 \quad (5)$$

$$\frac{\partial Q}{\partial p_h} = \sum_{k=0}^{t} \sum_{j=0}^{g} \beta_{ijk}[\frac{t-k+h}{p_h} - \frac{u+g-j}{1-p_m-p_h}] = 0 \quad (6)$$

Equation 6 $\Rightarrow p_h = M(1 - p_m)$, where

$$M = \frac{\sum_{k=0}^{t} \sum_{j=0}^{g} \beta_{ijk}(t-k+h)}{\sum_{k=0}^{t} \sum_{j=0}^{g} \beta_{ijk}(t+u+h+g-k-j)}.$$

Express p_h in terms of p_m in equation 5, we have

$$\sum_{k=0}^{t} \sum_{j=0}^{g} \beta_{ijk}[\frac{m+k+j}{p_m} - \frac{l}{1-p_m} - \frac{u+g-j}{(1-M)(1-p_m)}] = 0$$

$$\Rightarrow p_m^{(i+1)} = \frac{\sum_{k=0}^{t} \sum_{j=0}^{g} \beta_{ijk}(m+k+j)}{\sum_{k=0}^{t} \sum_{j=0}^{g} \beta_{ijk}(m+l+k+j+\frac{u+g-j}{1-M})} = \frac{m + \sum_{k=0}^{t} \sum_{j=0}^{g} \beta_{ijk}(k+j)}{m+l+h+g+u+t}, \quad (7)$$

$$p_h^{(i+1)} = M(1 - p_m^{(i+1)}).$$

	TAB 5×+ BS 5×				TAB 10×+ BS 10×				TAB 15×+ BS 15×			
%Overshoot sites	$p_m = 0.1$	0.3	0.5	0.7	$p_m = 0.1$	0.3	0.5	0.7	$p_m = 0.1$	0.3	0.5	0.7
$p_h = 0.1$	17.04%	5.46%	1.27%	0.12%	16.61%	2.65%	0.22%	0.00%	14.57%	1.25%	0.03%	0.00%
$p_h = 0.3$	25.16%	9.36%	1.94%		23.84%	5.28%	0.37%		22.00%	2.93%	0.08%	
$p_h = 0.5$	26.18%	8.26%			25.02%	4.39%			23.01%	2.31%		
$p_h = 0.7$	22.52%				21.60%				19.63%			
Reduced relative error of $\hat{p}_h$	$p_m = 0.1$	0.3	0.5	0.7	$p_m = 0.1$	0.3	0.5	0.7	$p_m = 0.1$	0.3	0.5	0.7
$p_h = 0.1$	57.93%	64.31%	73.48%	90.13%	23.58%	30.58%	36.69%	54.55%	18.47%	21.62%	22.72%	NA
$p_h = 0.3$	35.00%	39.05%	44.97%		17.63%	21.39%	24.20%		14.72%	15.90%	16.81%	
$p_h = 0.5$	25.55%	30.37%			14.44%	17.81%			11.85%	13.28%		
$p_h = 0.7$	16.24%				10.22%				8.84%			

Table 1. Percentage of overshoot sites observed in simulation data, and the reduced relative error of MLML $\hat{p}_h$ estimates (defined as the reduced absolute error by MLML compared with frequency estimates divided by the true level, averaged over overshoot sites) at different methylation levels, and coverages for combination of TAB-seq and BS-seq.

TAB + BS + oxBS	5×				10×			
%Overshoot sites	$p_m = 0.1$	0.3	0.5	0.7	$p_m = 0.1$	0.3	0.5	0.7
$p_h = 0.1$	68.55%	76.06%	76.83%	73.88%	78.14%	83.18%	84.30%	86.11%
$p_h = 0.3$	75.95%	78.58%	77.85%		82.77%	84.76%	86.88%	
$p_h = 0.5$	76.76%	77.71%			84.52%	86.80%		
$p_h = 0.7$	73.44%				86.17%			

Table 2. Proportion of overshoot sites when combining oxBS-seq, TAB-seq and BS-seq using 100,000 binomial simulations with the indicated p_m and p_h at 5× and 10× exact coverage.

TAB + BS + oxBS	$\hat{p}_m$				$\hat{p}_h$				$\hat{p}_u$			
Reduced relative error (5×)	$p_m = 0.1$	0.3	0.5	0.7	$p_m = 0.1$	0.3	0.5	0.7	$p_m = 0.1$	0.3	0.5	0.7
$p_h = 0.1$	47.43%	16.94%	13.88%	10.96%	47.31%	18.58%	14.08%	30.01%	5.79%	8.44%	9.54%	8.38%
$p_h = 0.3$	18.78%	11.29%	12.49%		17.37%	11.29%	12.49%		8.39%	6.38%	-1.06%	
$p_h = 0.5$	13.78%	12.98%			13.84%	12.62%			9.45%	-0.75%		
$p_h = 0.7$	29.65%				10.88 %				8.35%			
Reduced relative error (10×)	$p_m = 0.1$	0.3	0.5	0.7	$p_m = 0.1$	0.3	0.5	0.7	$p_m = 0.1$	0.3	0.5	0.7
$p_h = 0.1$	2.76%	8.02%	6.68%	4.65%	3.41%	-5.17%	-5.21%	-3.16%	4.01%	5.70%	7.47%	6.60%
$p_h = 0.3$	-5.54%	5.53%	5.77%		7.84%	5.65%	5.98%		5.77%	5.67%	3.21%	
$p_h = 0.5$	-5.74%	5.80%			6.66%	5.76%			7.39%	3.08%		
$p_h = 0.7$	-3.15%				4.62%				6.68%			

Table 3. MLML improves accuracy at overshoot sites: reduced relative error by MLML compared with frequency method at overshoot sites when 3 experiments are combined. Each value is based on 100,000 simulations, sampling reads for oxBS-seq, TAB-seq and BS-seq using binomials with the indicated p_m and p_h at 5× and 10× exact coverage.

	Percent Overshoot sites				Percent $\hat{p}_h$ error reduction				Percent $\hat{p}_u$ error reduction			
BS 5× + Tab 25×	$p_m = 0.1$	0.3	0.5	0.7	$p_m = 0.1$	0.3	0.5	0.7	$p_m = 0.1$	0.3	0.5	0.7
$p_h = 0.1$	31.72%	8.09%	1.19%	0.06%	7.55%	7.64%	7.69%	5.82%	10.39%	14.34%	20.13%	31.52%
$p_h = 0.3$	32.01%	9.40%	1.05%		3.37%	3.84%	4.32%		20.10%	27.90%	45.32%	
$p_h = 0.5$	30.88%	7.10%			2.17%	2.52%			32.53%	54.40%		
$p_h = 0.7$	25.91%				1.55%				60.77%			
BS 15× + Tab 15×	$p_m = 0.1$	0.3	0.5	0.7	$p_m = 0.1$	0.3	0.5	0.7	$p_m = 0.1$	0.3	0.5	0.7
$p_h = 0.1$	14.57%	1.25%	0.03%	0.00%	43.35%	38.71%	41.70%	NA	4.97%	7.46%	9.68%	NA
$p_h = 0.3$	22.00%	2.93%	0.08%		16.16%	17.14%	15.08%		7.66%	12.53 %	22.62%	
$p_h = 0.5$	23.01%	2.31%			9.89%	10.15%			11.57%	23.81%		
$p_h = 0.7$	19.63%				6.74%				20.74%			
BS 25× + Tab 5×	$p_m = 0.1$	0.3	0.5	0.7	$p_m = 0.1$	0.3	0.5	0.7	$p_m = 0.1$	0.3	0.5	0.7
$p_h = 0.1$	21.90%	4.20%	0.51%	0.02%	105.80%	95.09%	82.39%	70.83%	1.15%	2.20%	3.37%	7.08%
$p_h = 0.3$	29.34%	8.66%	1.50%		45.50%	37.60%	28.09%		1.56%	2.92%	5.62%	
$p_h = 0.5$	31.49%	9.29%			27.09%	19.94%			2.23%	5.00%		
$p_h = 0.7$	30.83%				16.52%				3.82%			

Table 4. Reduced relative error by MLML estimates TAB-seq and BS-seq using binomials with the indicated p_m and p_h at total 30× exact coverage.

	BS 5× + Tab 25×				BS 15× + Tab 15×				BS 25× + Tab 5×				BS 10× + Tab 10× + oxBS 10×			
MLML $\hat{p}_h$ MAE	$p_m = 0.1$	0.3	0.5	0.7	$p_m = 0.1$	0.3	0.5	0.7	$p_m = 0.1$	0.3	0.5	0.7	$p_m = 0.1$	0.3	0.5	0.7
$p_h = 0.1$	0.04	0.05	0.06	0.09	0.06	0.11	0.18	NA	0.08	0.23	0.40	0.59	0.07	0.08	0.08	0.07
$p_h = 0.3$	0.07	0.08	0.10		0.08	0.15	0.26		0.10	0.25	0.44		0.09	0.10	0.10	
$p_h = 0.5$	0.07	0.09			0.08	0.17			0.10	0.26			0.10	0.10		
$p_h = 0.7$	0.07				0.08				0.09				0.08			

Table 5. Mean absolute error (MAE) of MLML p_h estimates at overshoot sites for varying combinations of experiments at the same total coverage.
